# CT Angiography Analysis of Axillary Artery Diameter versus Common Femoral Artery Diameter: Implications for Axillary Approach for Transcatheter Aortic Valve Replacement in Patients with Hostile Aortoiliac Segment and Advanced Lung Disease

**DOI:** 10.1155/2016/3610705

**Published:** 2016-03-27

**Authors:** Rajiv Tayal, Humayun Iftikhar, Benjamin LeSar, Rahul Patel, Naveen Tyagi, Marc Cohen, Najam Wasty

**Affiliations:** Division of Cardiology, Newark Beth Israel Medical Center, Newark, NJ 07112, USA

## Abstract

*Objective.* The use of the axillary artery as an access site has lost favor in percutaneous intervention due to the success of these procedures from a radial or brachial alternative. However, these distal access points are unable to safely accommodate anything larger than a 7-French sheath. To date no studies exist describing the size of the axillary artery in relation to the common femoral artery in a patient population. We hypothesized that the axillary artery is of comparable size to the CFA in most patients and less frequently diseased.* Methods.* We retrospectively reviewed 110 CT scans of the thoracic and abdominal aorta done at our institution to rule out aortic dissection in which the right axillary artery, right CFA, left axillary artery, and left CFA were visualized. Images were then reconstructed using commercially available TeraRecon software and comparative measurements made of the axillary and femoral arteries.* Results.* In 96 patients with complete data, the mean sizes of the right and left axillary artery were slightly smaller than the left and right CFA. A direct comparison of the sizes of the axillary artery and CFA in the same patient yielded a mean difference of 1.69 mm ± 1.74. In all patients combined, the mean difference between the axillary artery and CFA was 1.88 mm on the right and 1.68 mm on the left. In 19 patients (19.8%), the axillary artery was of the same caliber as the associated CFA. In 8 of 96 patients (8.3%), the axillary artery was larger compared to the CFA.* Conclusions.* Although typically smaller, the axillary artery is often of comparable size to the CFA, significantly less frequently calcified or diseased, and in almost all observed cases large enough to accommodate a sheath with up to 18 French.

## 1. Introduction

Despite the presence of older surgical literature referencing its use in axillary-femoral bypass and access via surgical exposure for severe descending aortic disease, the use of the axillary artery (AxA) as an access site has lost favor in percutaneous intervention in large part due to the success of these procedures from a radial or brachial alternative [1–3]. However, these distal access points are typically unable to accommodate anything larger than a 6 or a 7 Fr sheath [4–6]. Many operators cite the AxA's proximity to the neurovascular bundle or uncertainty of its ability to reliably accommodate larger sheaths as their primary concerns in avoiding AxA access. Difficulties in effecting hemostasis given the arteries lack of compressibility on a bony structure further compound the reasons why the AxA is not routinely utilized as a percutaneous access site.

However, in the setting of hostile aortoiliofemoral (AIF) segments, we feel that the AxA should remain a viable consideration and may preclude the necessity for a surgical cut-down and exposure in many instances. Although use of the subclavian artery via an open surgical approach has been well described, a small number of isolated case reports regarding the percutaneous use of the AxA as an access site for transcatheter aortic valve replacement (TAVR), endovascular aneurysm repair (EVAR) of the aorta, and Impella supported high risk PCI have now been published [7]. To the best of our knowledge, to date no studies exist describing the in vivo size of the AxA in relation to the common femoral artery (CFA) in a general patient population. We hypothesized that the AxA will not only be of comparable size to the CFA in most patients but also be less frequently calcified or diseased and able to accommodate sheaths with up to an 18 Fr outer diameter in the vast majority of patients.

## 2. Methods

**Figure 1:**
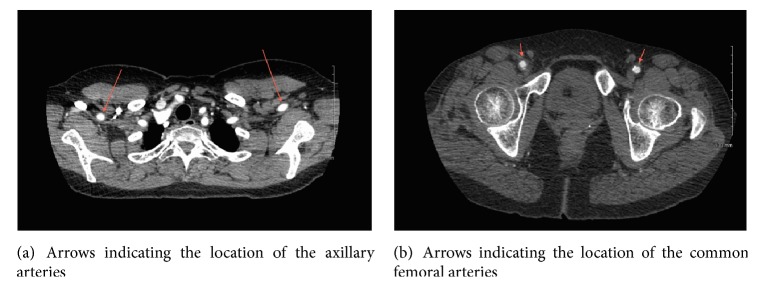
Contrasted enhanced CT scan images.

**Figure 2:**
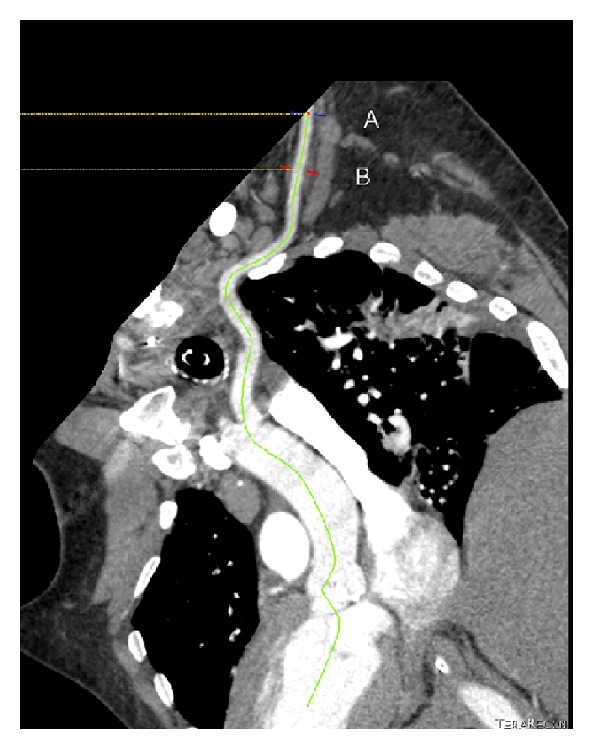
Curved multiplanar TeraRecon reconstruction of axillary artery with measurement at point B in axillary artery.

**Figure 3:**
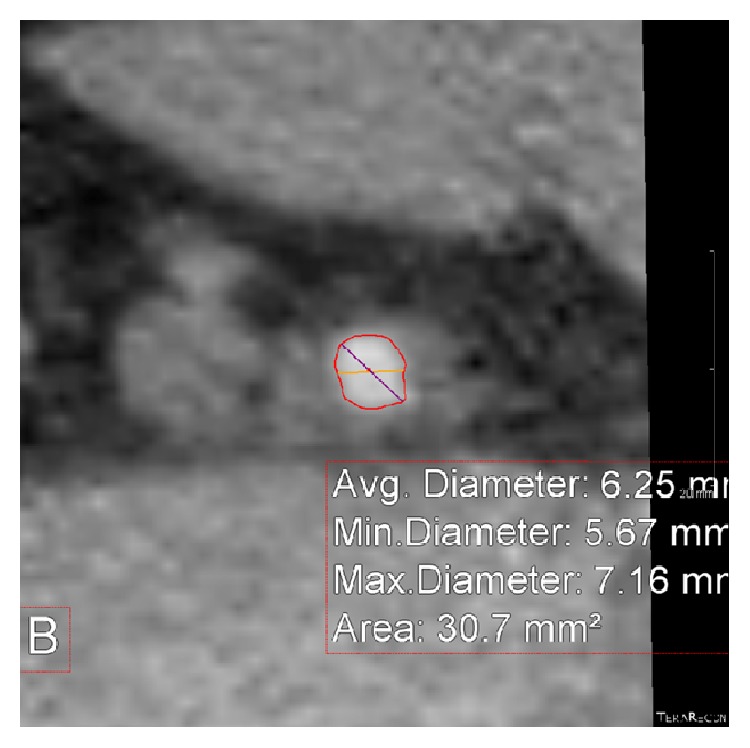
Cross-sectional image of the axillary artery with measurement of diameter at point B.

**Figure 4:**
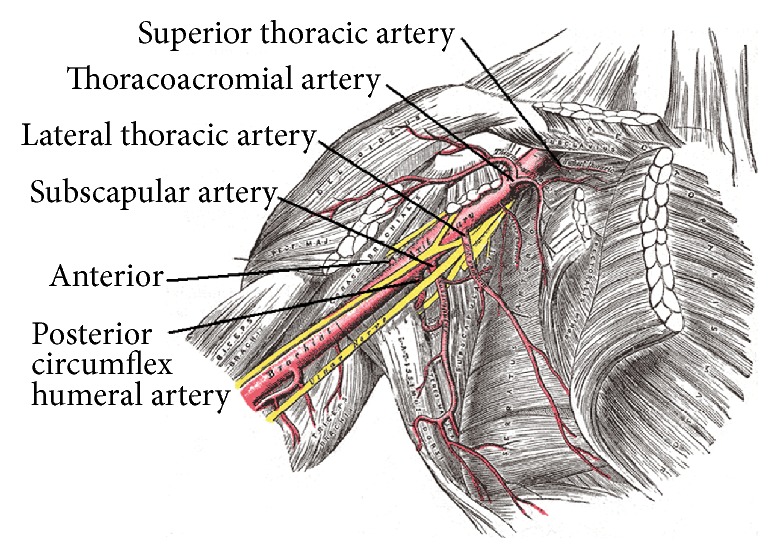
Review of axillary artery anatomy with preferred access site being medial to the lateral thoracic artery. Source: see [19]. Axillary artery is divided into three branches based on its location relative to the pectoralis minor muscle. The first, second, and third parts are medial, posterior, and lateral to the pectoralis minor muscle, respectively. The branches of the axillary artery supply the arm and the muscles of thorax and scapular region and this region is well collateralized providing circulation to the arm from arteries that arise from dorsal and suprascapular artery. Given the abundance of collateral circulation, axillary artery can be and is used for arterial cannulation during cardiac surgery, without endangering the circulation to the arm, especially if the access point is medial to the origin of the lateral thoracic artery, given its anastomosis with the intercostal and the internal mammary artery.

We retrospectively reviewed 110 contrast enhanced CT scans of the chest, abdomen, and pelvis performed at Newark Beth Israel Medical Center in which the right AxA, right CFA, left AxA, and left CFA were visualized (Figures 1(a) and 1(b)). Images were reconstructed utilizing commercially available TeraRecon software (Figure 2) and diameter measurements made of the CFA above the level of the bifurcation of the superficial femoral artery (SFA) and profunda artery but below the level of the inguinal ligament at which percutaneous arterial access is optimally achieved as well as at the first portion of the AxA just distal to the subclavian artery and prior to the origination of the lateral thoracic artery (Figures 3 and 4). The arterial diameter was calculated using TeraRecon; however, we manipulated the diameter to include the entire contrast filled vessel and to exclude the calcifications at the narrowest portion of the vessel. We manually drew the diameter and TeraRecon calculated the diameter minimum and maximum. Subsequently, grading of the calcified atherosclerosis was performed at all levels. Calcification severity was graded upon whether the calcification was mild, moderate, or severe. There was no direct calculation or measurement of calcification plaque. This was a gestalt on how much calcification was along the iliac arteries versus subclavian and axillary arteries. The actual detection of calcification was done via the CT scan and essentially the volume calculated by subtracting the narrowest vessel size from a normal vessel diameter. Hounsfield units would not be helpful and lengths of the calcifications would also be extremely difficult as they are usually several of all different sizes. Of the 110 CT scans reviewed, complete imaging and data were available in 96 patients. Patients were imaged in the supine position with hands above their head as per standard CT imaging protocol. Patients with inadequate visualization of any or all of the arteries due to artifact or inadequate contrast penetration were excluded from the study. Statistical calculations and analysis were performed using GraphPad software.

## 3. Results

**Figure 5:**
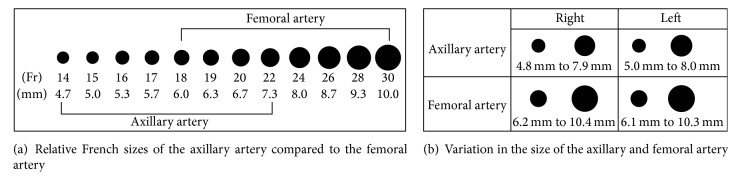
Diagrammatic representation of the size of the axillary arteries in comparison to common femoral arteries.

The demographics of the 96 patients with complete images and data available are listed in Table 1: 46.8% of patients were male, aged 61 ± 15.2 years, mean height of 168.2 ± 9.7 cm, weight 87.5 ± 29.5 kg, and BSA 1.95 ± 0.3. 83.4% were hypertensive and 45.9% had dyslipidemia, 25.7% diabetes mellitus, 29.3% coronary artery disease, 28.4% renal insufficiency, 11% end stage renal disease, and 13.8% peripheral vascular disease and 34.9% had a history of tobacco use. The mean sizes of the right and left AxAs were 6.38 ± 1.57 mm and 6.52 ± 1.52 mm, respectively, versus 8.26 ± 2.1 mm and 8.2 ± 2.09 mm for the right and left CFAs (Figure 5). A direct comparison of the sizes of the AxA and CFA in the same patient yielded a mean difference of 1.69 mm ± 1.74. In all patients combined, the mean difference between the AxA and CFA was 1.88 mm on the right and 1.68 mm on the left. Of all of the right and left AxAs studied, only 1.04–2.1% demonstrated calcification, versus 17.8–19.8% of the CFAs, a significantly lower percentage noted in the AxAs versus the CFAs (Table 2). Of the 96 patients studied, 19 had an AxA that was of the same caliber compared to their associated CFA representing 19.8% of all patients studied and 8 of 96 or 8.3% had one that was larger in size.

## 4. Discussion

Prior to the popularization of the radial approach in percutaneous intervention, access from the brachial artery initially via surgical cut-down and later percutaneously was commonplace [8]. However, the inability to safely and reliably accommodate anything larger than a 7 Fr sheath has proven to be the major drawback of these approaches. With the dramatic increase in the number of percutaneous transcatheter and endovascular procedures available that necessitate the use of large caliber sheaths including Impella supported high risk PCI (13-14 Fr, 4.33 to 4.67 mm), TAVR (14–26 Fr, 4.67 to 8.67 mm), and EVAR (9–22 Fr, 3 to 7.33 mm), evaluation for the presence of calcification and/or atherosclerotic plaques in the peripheral vasculature, including that of the aortoiliofemoral segment, is routinely performed [9, 10]. In many instances, severe atherosclerotic disease or calcification in these arterial segments precludes the use of the CFA all together. In fact, vascular access site related complications have remained the Achilles heel of many of these procedures and in patients undergoing TAVR have been reported to range from 6 to 14% and have been shown to affect patient survival [7, 10, 11]. In patients with the hostile AIF segments, alternative approaches include traditional open surgical repair, surgical exposure and cut-down of the common femoral or subclavian/axillary arteries [7, 12–14], or transapical (TA) and transaortic (TAo) access for TAVR. However in many instances these patients are at extremely high risk of standard or modified surgical intervention due to their clinical instability, significantly advanced age, or a myriad of comorbidities making the avoidance of general anesthesia, circulatory arrest, thoracotomies, mini sternotomies, and chest tubes preferable if possible.

The use of the subclavian and/or axillary artery has previously been well described as an alternative vascular access site after surgical cut-down in both Impella placement and TAVR [12–15]. Recently, several small case reports have described* percutaneous* use of the AxA for TAVR, Impella, and EVAR [15–18]. In fact, Schäfer et al. demonstrated a percutaneous access and closure technique of the AxA in 24 patients undergoing TAVR with both the Medtronic CoreValve and Edwards Sapien 3 valves without significant access site complications and a 100% procedural success rate [16]. The vascular complications experienced were safely handled with covered stent deployment. With regard to the potential complication of stroke, use of the left AxA minimizes the interference with the rest of the cerebral arteries, and between the Milan experience [15] and the Hamburg series [16], there was only 1 stroke attributed to the AxA route. While differences in the muscular and elastic composition of the CFA versus the AA have been described, to date, to the best of our knowledge, no studies exist comparing the diameter of the CFA to that of the AA [16]. Although it is typically smaller than the CFA, we suggest that in almost all patients the AA is an acceptable alternative access site for Impella placement, TAVR, and EVAR in the setting of a hostile AIF. Furthermore, our findings suggest it is also less prone to develop significant atherosclerosis and calcifications. We acknowledge that use of the percutaneous closure devices in the AxA is an “off-label” use.

## 5. Conclusions

Based on the results of our analysis, we suggest that the AA should be considered as an acceptable alternative access site for Impella placement, TAVR, and EVAR in the setting of a hostile AIF segment as we find it less often to be significantly diseased or calcified, although typically slightly smaller in overall caliber when compared to the CFA.

## Figures and Tables

**Table 1:** Patient demographics, *N* = 96.

Age (years)	61 ± 15.2
Sex	46.8% male
Height (cm)	168.2 ± 9.7
Weight (kg)	87.5 ± 29.5
Body Surface Area (BSA)	1.95 ± 0.3
Hypertension	83.4%
Dyslipidemia	45.9%
Diabetes mellitus	25.7%
Coronary artery disease	29.3%
Renal insufficiency	28.4%
End stage renal disease	11%
Peripheral vascular disease	13.8%
History of tobacco use	34.9%

**Table:** (a) Right axillary artery versus right common femoral artery

*N* = 96	R Ax	R CFA	Mean difference	*P*	95% CI
Mean (mm)	6.38	8.26	−1.88	<0.0001	−2.39 to −1.35
Median (mm)	6.2	8	−2		
Std. Dev. (mm)	1.57	2.1	1.76		
SEM	0.158	0.211			
Moderate-to-severe calcification (%)	1.04	17.8		<0.0001	

**Table:** (b) Left axillary artery versus left common femoral artery

*N* = 96	L Ax	L CFA	Mean difference	*P*	95% CI
Mean (mm)	6.52	8.2	−1.68	<0.0001	−2.21 to −1.17
Median (mm)	6.2	8	−2		
Std. Dev. (mm)	1.52	2.09	1.74		
SEM	0.156	0.214			
Moderate-to-severe calcification (%)	2.1	19.8		<0.0001	

## Abbreviations

AxA: Axillary artery
Fr: French
AIF: Aortoiliofemoral
TAVR: Transcatheter aortic valve replacement
CFA: Common femoral artery
EVAR: Endovascular aneurysm repair
TA: Transapical
TAo: Transaortic.
